# Exploring the mechanism of (-)-Epicatechin on premature ovarian insufficiency based on network pharmacology and experimental evaluation

**DOI:** 10.1042/BSR20203955

**Published:** 2021-02-12

**Authors:** Fei Yan, Qi Zhao, Huanpeng Gao, Xiaomei Wang, Ke Xu, Yishu Wang, Fuguo Han, Qingfei Liu, Yun Shi

**Affiliations:** 1 Dongzhimen Hospital, Beijing University of Chinese Medicine, Beijing, China; 2 School of Pharmaceutical Sciences, Tsinghua University, Beijing, China

**Keywords:** (-)-Epicatechin, network pharmacology, oxidative stress, premature ovarian insufficiency, protein kinase B

## Abstract

**Methods:** Relevant potential targets for EC were obtained based on Traditional Chinese Medicine System Pharmacology Database (TCMSP), a bioinformatics analysis tool for molecular mechanism of Traditional Chinese Medicine (BATMAN-TCM) and STITCH databases. The Online Mendelian Inheritance in Man (OMIM) and GeneCards databases were utilized to screen the known POI-related targets, while Cytoscape software was used for network construction and visualization. Then, the Gene Ontology (GO) and pathway enrichment analysis were carried out by the Database for Annotation, Visualization and Integrated Discovery (DAVID) database. Furthermore, KGN cells were performed to validate the predicted results in oxidative stress (OS) model, and antioxidant effect was examined.

**Results:** A total of 70 potential common targets for EC in the treatment of POI were obtained through network pharmacology. Metabolic process, response to stimulus and antioxidant activity occupied a leading position of Gene Ontology (GO) enrichment. Kyoto Encyclopedia of Genes and Genomes (KEGG) analysis indicated that PI3K/protein kinase B (AKT), TNF, estrogen, VEGF and MAPK signaling pathways were significantly enriched. In addition, cell experiments showed that EC exhibited antioxidant effects in an H_2_O_2_-mediated OS model in ovarian granulosa cells by regulating the expression of PI3K/AKT/nuclear factor erythroid 2-related factor 2 (Nrf2) signaling pathway and multiple downstream antioxidant enzymes.

**Conclusion:** EC could regulate multiple signaling pathways and several biological processes (BPs). EC had the ability to down-regulate elevated OS level through the PI3K/AKT/Nrf2 signaling pathway and represented a potential novel treatment for POI.

## Introduction

**Figure 1:**
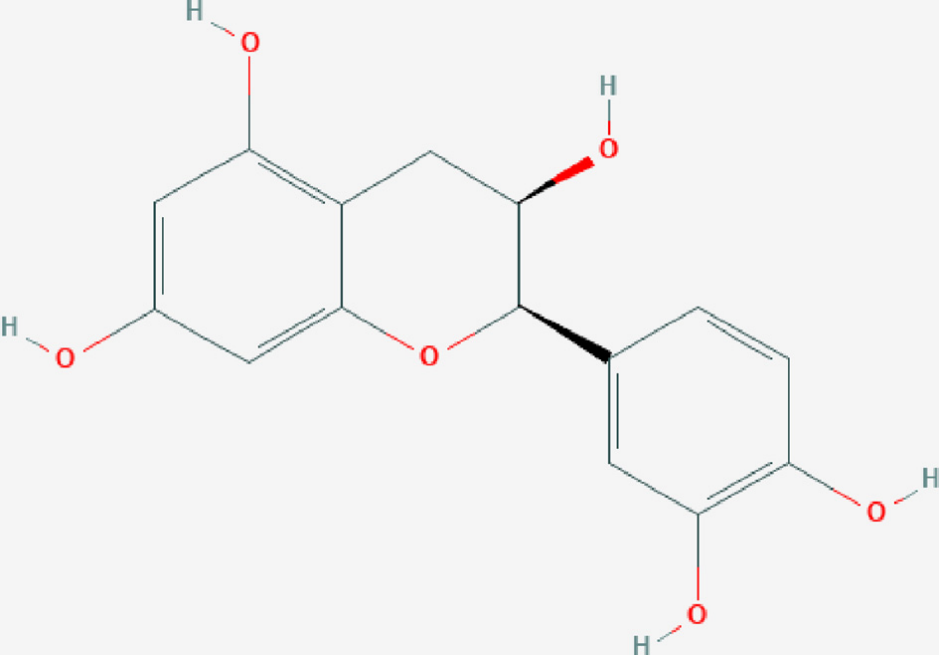
Molecular structure of (-)-Epicatechin

Premature ovarian insufficiency (POI) is a clinical syndrome defined as decrease, or even loss of ovarian function in women before the age of 40 years. It is characterized by menstrual disorders (amenorrhea or oligomenorrhea), hot flashes, night sweats and other perimenopausal symptoms, accompanied by elevated levels of gonadotropins and decreased estrogen concentrations [1]. POI is a common, spontaneous and heterogeneous disease [2], which can cause infertility, sexual dysfunction and represent an increased risk of osteoporosis, cardiovascular and neurodegenerative diseases [3]. Recent epidemiological studies have revealed that POI is becoming higher and younger in its attack rate. According to current data, POI affects approximately 1, 0.1 and 0.01% of women under the age of 40, 30 and 20 years, respectively, with the incidence varying slightly among different ethnicities [4]. The causes of POI are multifactorial, including hereditary disease, chromosomal defects, autoimmune disease, viral infection and iatrogenic factors. However, the underlying etiology remains unclear in majority of cases [5]. The importance of homeostasis of the ovarian microenvironment, especially the ovarian oxidative stress (OS) status, is a novel finding that has received more and more attention from researchers worldwide [6]. OS is also considered to be an important pathological factor to cause POI [7,8]. The current guidelines for the treatment of POI in modern medicine including hormone replacement therapy (HRT) and assisted reproductive technology (ART), aim to maintain secondary sexual characteristics and meet the needs of patients with fertility requirements. However, these therapies have side effects, such as increased risk of breast cancer and endometrial carcinoma, and the induction of ovarian hyperstimulation syndrome (OHSS) [9]. Therefore, a more effective and specific treatment for POI is needed urgently.

(-)-Epicatechin (EC) is one of the most abundant naturally occurring polyphenol compounds found in the human diet and is commonly found in plants as a secondary metabolite [10]. It is the primary form of flavan-3-alcohol, which commonly exists in cocoa, tea, apple and catechus [11]. As shown in Figure 1, the molecular structure of EC is composed of two aromatic rings and one oxygen-containing heterocyclic ring. This stereochemical configuration makes it easier to be absorbed orally compared with other monomeric forms in the catechin category [12]. Furthermore, the presence of epigallate and a hydroxyl group, as well as the number and position of its double bonds, give it a unique pharmacological profile [13]. It has been shown that EC has powerful antioxidant and anti-inflammatory effects and is considered to be one of the best naturally occurring compounds for the treatment and prevention of various related diseases [14]. EC itself and EC-rich foods have been demonstrated to have significant clinical efficacy in the treatment of cardio-cerebrovascular diseases, the prevention of metabolic disorders, the enhancement of muscle strength and the maintenance of nerve function in a number of clinical trials [15,16]. It has also been used to treat chronic inflammation independently and applied as an adjuvant therapy in combination with dexamethasone or any other drugs [17]. However, so far, no studies have been performed about the effect of EC on the improvement of reproductive function and protection of the ovary, as current research has mainly focused its effects on other diseases.

Network pharmacology, based on the theory of systems biology and high-throughput research, can be used to construct a ‘component–target–pathway’ network and demonstrate effects on a particular disease by a specific drug systematically and comprehensively [18]. Network pharmacology has been successfully used to reveal the molecular pharmacological effects of drugs and predict their therapeutic targets [19,20].

In the present study, the relevant targets, biological process (BP) and signaling pathways of EC were systematically explored and predicted by network pharmacology. Furthermore, an OS cell model of ovarian granulosa cell was constructed to identify the nature of this antioxidant effect produced by EC.

## Materials and methods

### Targets related to EC

Relevant potential targets for EC were obtained using Traditional Chinese Medicine System Pharmacology Database (TCMSP, http://lsp.nwu.edu.cn/tcmsp.php), a Bioinformatics Analysis Tool for Molecular Mechanism of Traditional Chinese Medicine (BATMAN-TCM, http://bionet.ncpsb.org/batman-tcm/) and STITCH (http://stitch.embl.de/) databases. TCMSP and BATMAN provide comprehensive pharmacological information involving more than 20,000 compounds, and they can also predict protein targets related to the compound, facilitating a better understanding of their pharmacological effects. STITCH database is a platform for searching the interaction between known and predicted compounds and proteins. The relationship between a compound and a protein is evaluated by a 10-point confidence score (10 = most confident). A confidence score of ≥7 is generally defined as a good compound-related target. All targets were uploaded to Uniprot (http://www.uniprot.org/) after duplication, where they could be standardized named for accurate analysis.

### Targets related to POI

Known POI-related targets were screened in the following two databases: Online Mendelian Inheritance in Man (OMIM) database (https://omim.org) and GeneCards database (https://www.genecards.org/). OMIM database is a constantly updated database consisting of Human Mendelian genetic diseases, which focuses on the relationship between human genetic variation and phenotypic traits. GeneCards database can comprehensively display the association between genotype and phenotype, gene interaction, signaling pathways and clinical significance.

### Protein–protein interaction network construction

Common targets for EC and POI were imported into STRING database (https://string-db.org/, ver.11.0) for protein–protein interaction (PPI) analysis. The filter condition was selected as ‘*Homo sapiens*’, and the minimum interaction score was set at 0.700. A PPI network graph was finally constructed, in which the ‘node’ information was the target and the relationship between nodes was represented by ‘edge’. Finally, the results of PPI were imported into Cytoscape software (Version 3.7.2) to create a visual display. According to the Cytohubba plug-in MCC algorithm in the Cytoscape software, the top eight proteins with highest core degree were analyzed and finally the potential core target could then be predicted.

### Gene Ontology and Kyoto Encyclopedia of Genes and Genomes enrichment analyses

The Database for Annotation, Visualization and Integrated Discovery (DAVID) database (https://david.ncifcrf.gov/) was used to analyze Gene Ontology (GO) function and Kyoto Encyclopedia of Genes and Genomes (KEGG) pathway enrichment of the common target proteins. GO function analysis is mainly used to describe the type of function an identified gene may possess, including cell function, molecular function (MF) and biological function. KEGG enrichment analysis is used to obtain the potential signaling pathways enriched of EC against POI.

### Drugs and reagents

EC (CAS No: 490-46-0, Lot No: MUST-19043012) was purchased from Chengdu Must Bio-Technology (Chengdu, China) and dissolved in DMSO at a stock concentration of 2 mg/ml. To avoid potential cytotoxicity, the final concentration of DMSO was used at <0.1%.

H_2_O_2_ (3%, W/V) was purchased from Shandong Lierkang Medical Technology Co. Ltd (Shandong, China) and stored at 4°C in the dark. Fetal bovine serum (FBS), DMEM/F12 medium and penicillin/streptomycin were purchased from Gibco (Grand Island, NY, U.S.A.). A cell counting kit 8 (CCK-8) was purchased from Dojindo Laboratories (Tokyo, Japan). Superoxide dismutase (SOD), reduced glutathione (GSH) and oxidized glutathione (GSSG) assay kits were purchased from Nanjing Jiancheng Co. Ltd (Nanjing, China). Total RNA Extraction Kit (DNase I), mRNA cDNA Synthesis Kit and mRNA/lncRNA qPCR Kit/RNA Loading Buffer (5×) were purchased from GenePool. eNOS, PI3 kinase p85 α (PI3K), nuclear factor erythroid 2-related factor 2 (Nrf2), heme oxygenase 1 (HO-1), actin antibody, goat anti-mouse IgG and goat anti-rabbit IgG were all purchased from Abcam (Cambridge, MA, U.S.A.), and protein kinase B (AKT/PKB) antibody was purchased from CST.

### Cell culture

The ovarian granulosa cell line, KGN, was obtained commercially from Beijing Beina Chuanglian Biotechnology Institute (Beijing, China). The cells were cultured in DMEM/F12 medium supplemented with 10% FBS, 100 U/ml penicillin and 100 mg/ml streptomycin and maintained in a humidified chamber at 37°C under 5% CO_2_ atmosphere.

### Cell viability assay

KGN cells were seeded into 96-well culture plates at a density of 4 × 10^4^ cells/ml for 24 h and then exposed to different concentrations of H_2_O_2_ and EC, respectively. After 24 h of incubation, CCK-8 reagent was added and the plates were placed on a plate shaker for 1 min to ensure optimal mixing. After incubation for 2.5 h, the absorbance was measured at 450 nm using a microplate reader. The survival rate of the cells was calculated according to the following formula: Survival rate = treatment group/control group × 100%. Each experiment was repeated three times.

### Modeling and intervention

The cells were exposed to H_2_O_2_ for 24 h to stimulate oxidative injury and then the medium was removed. EC was added to the cells at three different concentrations: 100, 200 and 300 μM and the cells were cultured for 24 h.

### Antioxidant activity assay

The activities of the antioxidant enzyme (SOD) and the antioxidant substrates (GSH and GSSG) in all groups were evaluated by a commercially available assay kit. All experimental protocols were carried out according to the manufacturer's instructions.

### RNA extraction and real-time PCR

Total RNA was extracted from each group using Trizol reagent and its purity and concentration were determined. Next, the total RNA was reverse transcribed into cDNA according to the manufacturer's instructions. PCR was then performed using a real-time PCR Master Mix (SYBR Green) kit, and the relevant cycling conditions were set on the PCR machine for the amplification of PI3K, AKT, Nrf2, HO-1, NADH quinone dehydrogenase 1 (NQO1), nicotinamide adenine dinucleotide phosphate (NADPH) and β-actin. Moreover, the *C*_t_ values from the internal reference group and each experimental group were recorded. The relative expression of target genes was then calculated using the 2^ΔΔ*C*_t_^ method and normalized to β-actin. The primer sequences used are indicated below (Table 1).

**Table 1:** Primer design

Gene name	Primer sequence	Length (bp)
*PI3Ka*	(F) TTGCTGTTCGGTGCTTGGA	277
	(R) ACTTGCCTATTCAGGTGCTTCA	
*AKT*	(F) TGGCACCTTCATTGGCTACA	220
	(R) AGTCTGGATGGCGGTTGTC	
*Nrf2*	(F) ATTCCTTCAGCAGCATCCTCTC	86
	(R) ATCTGTGTTGACTGTGGCATCT	
*Hmox1*	(F) CCAGCAACAAAGTGCAAGATTC	105
	(R) TGAGTGTAAGGACCCATCGGAG	
*NQO1*	(F) GAGCGAGTGTTCATAGGAGAGT	217
	(R) TCAGTTGAGGTTCTAAGACTTGGA	
*NADPH*	(F) ACTACTATCTATGCTGAGACTGGTT	137
	(R) CCTGGTTGAATCACATTGAATCG	
*Actin*	(F) ACTTAGTTGCGTTACACCCTT	155
	(R) GTCACCTTCACCGTTCCA	

### Protein extraction and Western blot

Cells were harvested and washed twice with pre-chilled PBS, and the total protein was extracted using lysis buffer. Cell debris was centrifuged at 12,000 rpm for 15 min at 4°C, then the supernatants were collected and the protein concentration was determined using a BCA protein assay. After that, the samples were subjected to sodium dodecyl sulfate/polyacrylamide gel electrophoresis (SDS/PAGE) and the resulting protein bands were transferred onto a transfer membrane and blocked. Next, the following primary antibodies (PI3K) (1:1,000 dilution) and (AKT, Nrf2, HO-1, eNOS) (1:500 dilution), were added to the blocked membranes and incubated at 4°C overnight. The next day, the membranes were washed with PBS and then secondary antibody was applied (1:5,000 dilution). Finally, the membranes were washed again and then subjected to ECL. Protein quantitation of the developed bands was performed using QuantityOne software (ver.4.6.2, Bio-Rad, Hercules, California, U.S.A.) and the relative quantity of each protein was expressed as the gray value ratio of target protein to the internal reference band β-actin.

### Statistical analysis

SPSS 25.0 software was used for statistical analysis. The experimental results were presented as means ± standard deviation (SD) from three independent repetitions, and Student's *t* test was used to evaluate differences between two groups and a value of *P*<0.05 represented statistical significance.

## Results

### Potential EC targets related to POI treatment

One hundred and twenty-six potential targets of EC were screened in TCMSP, BATMAN and STITCH databases. A total of 7417 targets of POI were obtained from OMIM and GeneCards databases after deletion of overlaps. By exploring the intersects through two datasets, 70 potential common targets for EC in the treatment of POI were obtained (Table 2).

**Table 2:** Seventy potential common targets for EC in the treatment of POI

Target gene	Target protein	Target gene	Target protein
*ABCG2*	ATP-binding cassette subfamily G member 2	*ABCC1*	Multidrug resistance-associated protein 1
*ABCC2*	Canalicular multispecific organic anion transporter 1	*ACE*	Angiotensin-converting enzyme
*ACTB*	Actin, cytoplasmic 1	*AHR*	Aryl hydrocarbon receptor
*AKT1*	RAC-α serine/threonine-protein kinase	*ALB*	Serum albumin
*ALOX5*	Arachidonate 5-lipoxygenase	*APOB*	Apolipoprotein B-100
*ATP5A1*	ATP synthase subunit α, mitochondrial	*CALM*	Calmodulin 3 (phosphorylase kinase, Δ)
*CASP3*	Caspase-3	*CCL2*	C–C motif chemokine 2
*CDK6*	Cyclin-dependent kinase 6	*CEBPB*	CCAAT/enhancer-binding protein β
*CNR1*	Cannabinoid receptor 1	*CNR2*	Cannabinoid receptor 2
*COMT*	Catechol O-methyltransferase	*CREB1*	Cyclic AMP-responsive element-binding protein 1
*CSF2*	Granulocyte-macrophage colony-stimulating factor	*CSNK2A1*	Casein kinase II subunit α
*CYP19A1*	Aromatase	*CYP1A2*	Cytochrome P450 1A2
*CYP1B1*	Cytochrome P450 1B1	*CYP2C8*	Cytochrome P450 2C8
*DNMT1*	DNA (cytosine-5)-methyltransferase 1	*DRD1*	D(1A) dopamine receptor
*ESR1*	Estrogen receptor	*ESR2*	Estrogen receptor β
*ESRRB*	Steroid hormone receptor ERR2	*GCLC*	Glutamate-cysteine ligase catalytic subunit
*GRIN1*	Glutamate receptor ionotropic, NMDA 1	*GSS*	Glutathione synthetase
*HAS2*	Hyaluronan synthase 2	*HCK*	Tyrosine-protein kinase HCK
*HMOX1*	Heme oxygenase 1	*HSP90AA1*	Heat shock protein HSP 90-α
*HSPA2*	Heat shock-related 70-kDa protein 2	*IL1A*	Interleukin-1 α
*IL2*	Interleukin-2	*IL6*	Interleukin-6
*JAK1*	Tyrosine-protein kinase JAK1	*JUN*	Transcription factor AP-1
*KLK2*	Kallikrein-2	*LRTOMT*	Transmembrane O-methyltransferase
*MAOA*	Amine oxidase [flavin-containing] A	*NCOA1*	Nuclear receptor co-activator 1
*NR1I2*	Nuclear receptor subfamily 1 group I member 2	*PIK3CG*	Phosphatidylinositol 4,5-bisphosphate 3-kinase catalytic subunit γ isoform
*PLAT*	Tissue-type plasminogen activator	*PLAU*	Urokinase-type plasminogen activator
*PON1*	Serum paraoxonase/arylesterase 1	*POR*	NADPH–cytochrome P450 reductase
*PRKCA*	Protein kinase C α type	*PRKCB*	Protein kinase C β type
*PTGS1*	Prostaglandin G/H synthase 1	*PTGS2*	Prostaglandin G/H synthase 2
*RUVBL2*	RuvB-like 2	*RXRA*	Retinoic acid receptor RXR-α
*SHBG*	Sex hormone-binding globulin	*SLC16A1*	Monocarboxylate transporter 1
*SOAT1*	Sterol O-acyltransferase 1	*SRC*	Proto-oncogene tyrosine-protein kinase Src
*SULT1A1*	Sulfotransferase 1A1	*TNF*	Tumor necrosis factor
*TOP2A*	DNA topoisomerase 2-α	*TTR*	Transthyretin
*UBA1*	Ubiquitin-like modifier-activating enzyme 1	*VEGFA*	Vascular endothelial growth factor A

### Construction of PPI network

**Figure 2:**
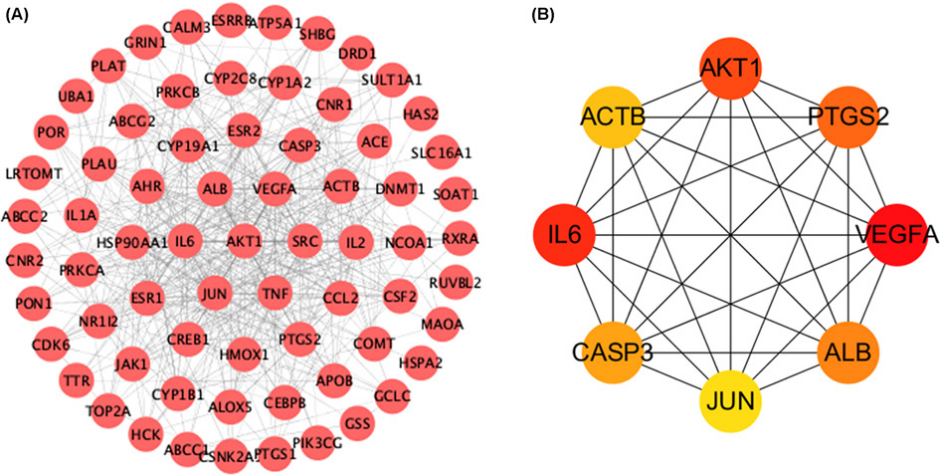
PPI network and top eight hub targets (**A**) PPI network related to EC in treatment of POI. (**B**) The top eight hub gene network of EC in treatment of POI by the MCC algorithm. The deeper the color is, the more important it is in the network.

Common targets were imported into STRING and the results were imported into Cytoscape to display a visual PPI network. There were 70 nodes and 492 edges in the graph. According to the MCC algorithm in the Cytohubba plug-in, the top eight potential candidate genes were obtained, and these were: *AKT1, ACTB, ALB, JUN, PTGS2, VEGFA, CASP3* and *IL6* (Figure 2).

### GO enrichment analysis

**Figure 3:**
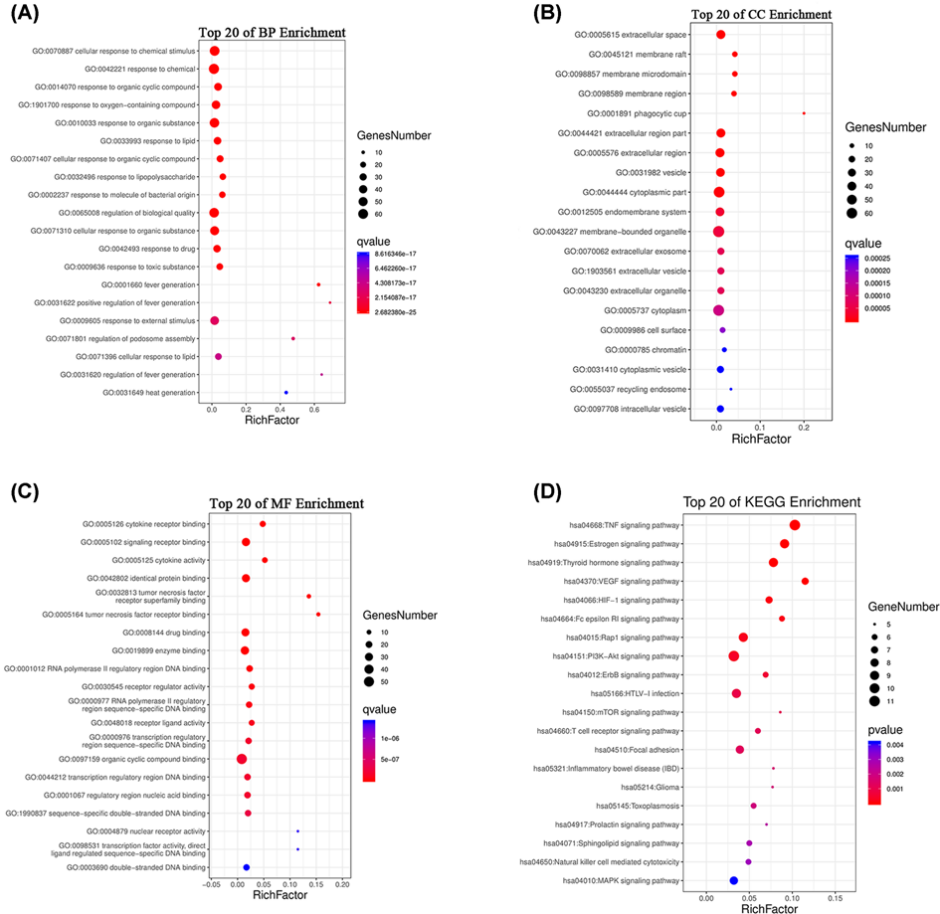
GO and KEGG analysis of targets (**A**) BP. (**B**) CC. (**C**) MF. (**D**) KEGG pathway. The size of the dots corresponds to the number of genes annotated in the entry, and the color of the dots corresponds to the corrected *P*-value.

To better understand the potential pharmacological activities of EC in the treatment of POI, the DAVID database was used to perform GO and KEGG enrichment analyses on the common targets. The results of GO analysis suggested that the BP was significantly enriched in cellular process, metabolic process, biological regulation and response to stimulus. Cell component (CC) was principally enriched in extracellular space, membrane raft, membrane microdomain and membrane region. The MF was enriched in binding, catalytic activity, molecular function regulation and antioxidant activity (Figure 3A–C).

### KEGG enrichment analysis

**Figure 4:**
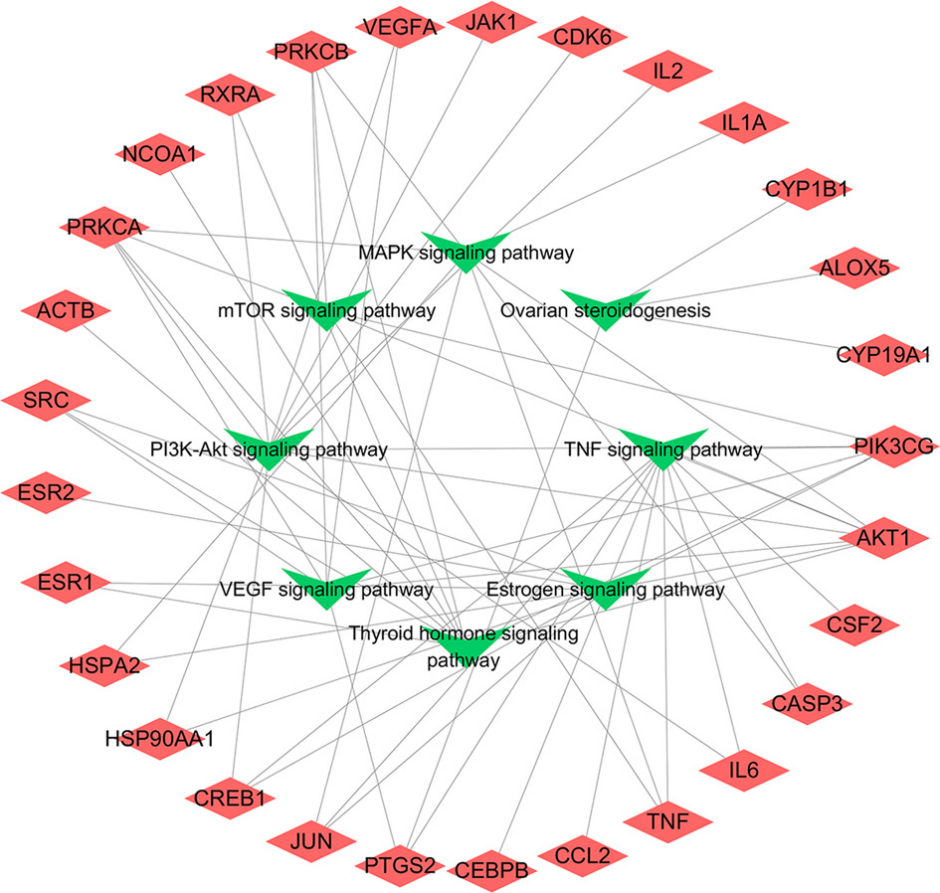
Target–pathway network The red diamonds represent common targets and the green triangles represent enriched pathways.

A total of 90 signaling pathways were obtained by KEGG enrichment analysis, and the top 20 pathways with high significance were selected to be displayed in combination with a literature search (Figure 3 and Table 3). The signaling pathways closely related to POI included PI3K/AKT, TNF, estrogen, thyroid hormone, VEGF, mTOR and MAPK signaling pathway. According to the results of our KEGG pathway enrichment, a potential target–pathway network map had been constructed and visualized with Cytoscape software (Figure 4).

**Table 3:** KEGG pathway analysis based on EC–POI network (top 20 with *P*-value)

ID	Pathway	Genes	*n*	*P*-value
hsa04668	TNF signaling pathway	*PIK3CG, AKT1, CSF2, CASP3, IL6, TNF, CCL2, CEBPB, PTGS2, JUN, CREB1*	11	1.06 × 10^−8^
hsa04915	Estrogen signaling pathway	*PIK3CG, AKT1, HSP90AA1, HSPA2, JUN, CREB1, ESR1, ESR2, SRC*	9	1.11 × 10^−6^
hsa04919	Thyroid hormone signaling pathway	*ACTB, PRKCA, PIK3CG, AKT1, NCOA1, RXRA, ESR1, SRC, PRKCB*	9	3.48 × 10^−6^
hsa04370	VEGF signaling pathway	*PRKCA, PIK3CG, AKT1, PTGS2, VEGFA, SRC, PRKCB*	7	8.70 × 10^−6^
hsa04066	HIF-1 signaling pathway	*PRKCA, PIK3CG, AKT1, IL6, HMOX1, VEGFA, PRKCB*	7	1.17 × 10^−4^
hsa04664	Fcϵ RI signaling pathway	*PRKCA, PIK3CG, AKT1, CSF2, TNF, PRKCB*	6	2.10 × 10^−4^
hsa04015	Rap1 signaling pathway	*ACTB, PRKCA, PIK3CG, AKT1, CNR1, VEGFA, GRIN1, SRC, PRKCB*	9	2.66 × 10^−4^
hsa04151	PI3K-Akt signaling pathway	*PRKCA, PIK3CG, AKT1, IL6, HSP90AA1, RXRA, CREB1, VEGFA, JAK1, CDK6, IL2*	11	3.98 × 10^−4^
hsa04012	ErbB signaling pathway	*PRKCA, PIK3CG, AKT1, JUN, SRC, PRKCB*	6	6.63 × 10^−4^
hsa05166	HTLV-I infection	*PIK3CG, AKT1, CSF2, IL6, TNF, JUN, CREB1, JAK1, IL2*	9	9.45 × 10^−4^
hsa04150	mTOR signaling pathway	*PRKCA, PIK3CG, AKT1, TNF, PRKCB*	5	1.21 × 10^−3^
hsa04660	T cell receptor signaling pathway	*PIK3CG, AKT1, CSF2, TNF, JUN, IL2*	6	1.24 × 10^−3^
hsa04510	Focal adhesion	*ACTB, PRKCA, PIK3CG, AKT1, JUN, VEGFA, SRC, PRKCB*	8	1.30 × 10^−3^
hsa05321	Inflammatory bowel disease (IBD)	*IL6, TNF, JUN, IL1A, IL*2	5	1.74 × 10^−3^
hsa05214	Glioma	*PRKCA, PIK3CG, AKT1, CDK6, PRKCB*	5	1.85 × 10^−3^
hsa05145	Toxoplasmosis	*AKT1, CASP3, TNF, HSPA2, JAK1, ALOX5*	6	1.91 × 10^−3^
hsa04917	Prolactin signaling pathway	*PIK3CG, AKT1, ESR1, ESR2, SRC*	5	2.55 × 10^−3^
hsa04071	Sphingolipid signaling pathway	*PRKCA, PIK3CG, AKT1, TNF, ABCC1, PRKCB*	6	2.79 × 10^−3^
hsa04650	Natural killer cell-mediated cytotoxicity	*PRKCA, PIK3CG, CSF2, CASP3, TNF, PRKCB*	6	3.00 × 10^−3^
hsa04010	MAPK signaling pathway	*PRKCA, AKT1, CASP3, TNF, HSPA2, JUN, IL1A, PRKCB*	8	4.16 × 10^−3^

### Cell viability assay

**Figure 5:**
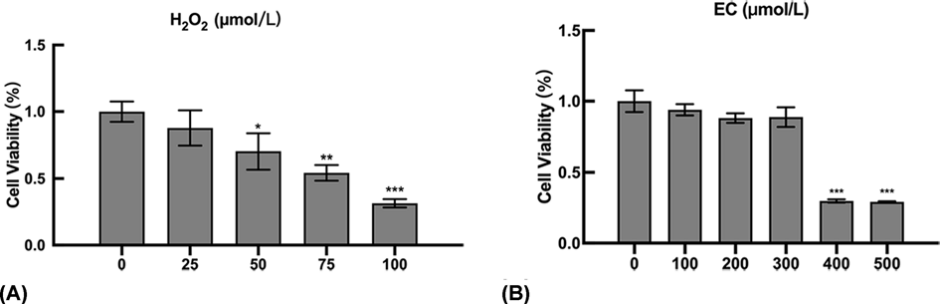
Effect of H_2_O_2_ and EC on cell viability using the CCK-8 assay (**A**) Cell viability of H_2_O_2_-induced KGN cells after 24 h. (**B**) Cell viability of EC-stimulated KGN cells after 24 h. *t* test, *P<0.05, **P<0.01, ***P<0.001 vs the control group.

H_2_O_2_ was used to stimulate OS in KGN cells, as has been widely reported in literature. The cell viability of KGN was found to be gradually decreased as the concentration of H_2_O_2_ increased. When an H_2_O_2_ concentration of 50 μM was used, the viability of the KGN cells was 70% (Figure 5A). As a result, this concentration was selected for all subsequent experiments.

To evaluate the potential toxicity of EC to KGN cells, cultures were incubated with concentrations ranging from 100 to 500 μM for 24 h (Figure 5B). Results from this found that at concentrations of EC between 100 and 300 μM, no significant changes were seen in cell viability. However, cytotoxicity from EC started to appear at 400 and 500 μM (*P*<0.001). Therefore, EC concentrations of 100, 200 and 300 μM were chosen as safe concentrations and were used in the remaining experiments.

### Determination of antioxidant enzymes and substrates

**Figure 6:**
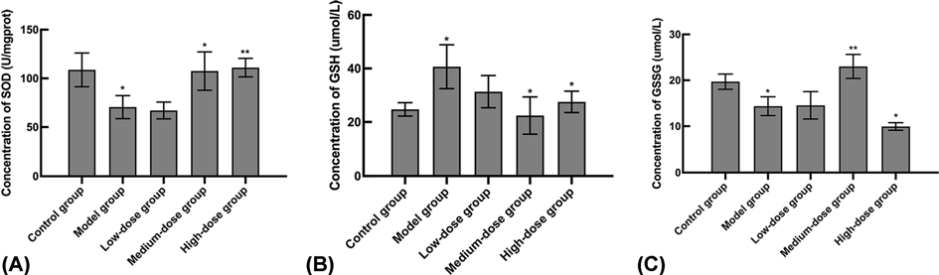
The concentration levels of SOD, GSH and GSSG (**A**) SOD. (**B**) GSH. (**C**) GSSG. *t* test, *P<0.05, **P<0.01 vs the model group.

The activity of SOD was significantly decreased in cells exposed to H_2_O_2_ (*P*<0.05), whereas the observed H_2_O_2_-induced injury was mitigated by an increasing concentration of EC (Figure 6A). This finding became highly significant in the medium and high dose groups (*P*<0.05, *P*<0.01). In addition, the activities of GSH and GSSG in all groups were evaluated (Figure 6B,C) and in the model group, GSH was significantly increased whereas GSSG was significantly decreased (*P*<0.05, *P*<0.05). Interestingly, when the medium concentration of EC was used, it exhibited its greatest efficacy at both the suppression of GSH expression and the enhancement of GSSG expression (*P*<0.05, *P*<0.01).

### Real-time PCR

**Figure 7:**
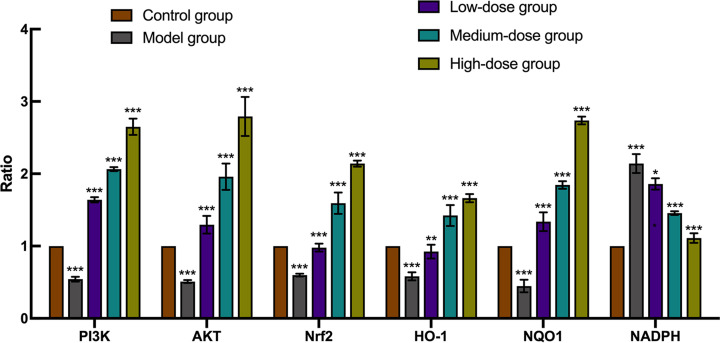
The mRNA analysis of PI3K, AKT, Nrf2, HO-1, NQO1, NADPH detected by real-time PCR *t* test, *P<0.05, **P<0.01, ***P<0.001 vs the model group.

Real-time quantitative PCR analysis was used to evaluate the mRNA levels of members of the PI3K/AKT/Nrf2 signaling pathway and its downstream antioxidant protein products (Figure 7). Results indicated that all of the mRNA expression levels for the above genes had significant differences in model group, when compared with the control group (*P*<0.001). Furthermore, all of the EC treated groups exhibited higher PI3K, AKT, Nrf2, HO-1 and NQO1 but lower NADPH expression levels when compared with the model group (*P*<0.05, *P*<0.01, *P*<0.001). In the high-dose (300 μM) EC category, the expression levels of PI3K, AKT, Nrf2, HO-1 and NQO1 genes peaked, whereas NADPH reached its lowest value.

### Western blot

**Figure 8:**
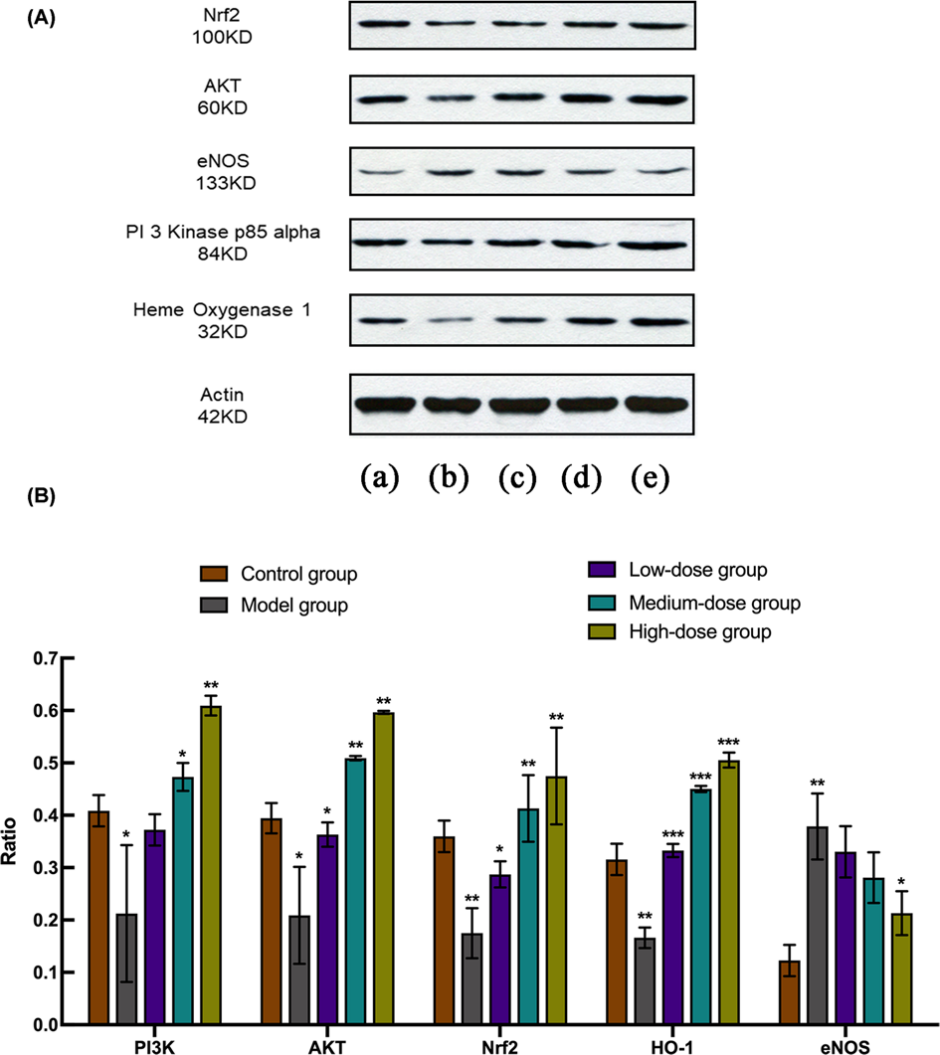
The expression analysis of PI3K, AKT, Nrf2, HO-1, eNOS detected by Western blot (**A**) The results of Western blot. (**B**) The relative protein expression of PI3K, AKT, Nrf2, HO-1and eNOS. *t* test, *P<0.05, **P<0.01, ***P<0.001 vs the model group. (a) Control group. (b) Model group. (c) Low-dose group. (d) Medium-dose group. (e) High-dose group.

Protein levels of AKT-related genes and OS marker proteins, including PI3K, AKT, Nrf2, HO-1 and eNOS, were successfully detected by Western blot (Figure 8). The concentrations of these proteins in model group all had significant differences, when compared with the control group (*P*<0.05). However, eNOS represented the only protein which was found to be up-regulated (*P*<0.01). After EC application at different doses, all protein expression levels were significantly reversed (*P*<0.05), whereas the level of PI3K in the low-dose group, and eNOS in low- and medium-dose groups showed no significant differences when compared with the model group.

## Discussion

POI is a complex disease with multiple pathological mechanisms leading to premature ovarian decline or even ovarian failure. Increasingly, research attention has been focused on the influence of the ovarian microenvironment in particular OS. OS, which refers to a state in which reactive oxygen species in tissues or organs are excessively elevated, is considered to be a potential cause of POI. Significantly, levels of OS in ovarian tissue can not only lead to abnormal activation of original follicles, but also induce the growth of follicles and their entry into the atresia orbit, resulting in abnormal follicular grow and discharge, ultimately leading to POI [21].

EC, as a polyphenolic compound with significant antioxidant effect, has been extensively studied in pharmacological experiments. EC has been demonstrated to have excellent antioxidant properties in a wealth of *in vivo* and *in vitro* experiments such as bovine spermatozoa, primary endothelial cells and senile mice [22–24]. Its metabolites can accumulate in the body and provide a wide range of beneficial effects on tissues and organs [25]. For this reason, EC has been considered to be one of the best available natural products for treating and preventing various chronic diseases.

The present study was performed to explore the antioxidant mechanism of EC in the treatment of POI by coupling network pharmacology and *in vitro* cell assays. We conducted network pharmacological database analysis of EC treatment in POI, constructed a PPI network, searched for core genes and carried out pathway enrichment analysis. Utilizing a literature-based investigation, we found that AKT1, IL6 and CASP3 in core genes and PI3K/AKT, VEGF and MAPK pathways in concentrated pathways were closely related to OS. AKT, a serine/threonine kinase, also known as PKB, is a crucial growth regulator in many signaling pathways [26]. Specific toxins such as nicotine and ochratoxin A can reduce AKT expression level in granulosa cells and original follicles, thereby affecting ovarian reserve function [27]. Antioxidants such as resveratrol and curcumin can up-regulate the expression of AKT and reduce OS level in ovarian tissue in animal models of POI, thereby regulating apoptosis in granulosa cells and improving ovarian function [28,29]. IL-6 is a typical immune response regulatory factor and its expression is closely related to levels of OS damage in cells or tissues, regardless of premature ovarian failure (POF) [30], polycystic ovarian syndrome (PCOS) [31], or ovarian damage caused by environmental pollutants [32]. CASP3 represents a terminal executioner caspase and can be activated by various signals including OS, which can cause DNA breakage and granulosa cell apoptosis, ultimately leading follicular atresia and damage to ovarian function [33]. The signaling cascade mediated by VEGF/VEGFR2 can principally regulate the proliferation, migration and survival of vascular endothelial cells, improve vascular endothelial permeability and maintain an adequate blood supply [34]. Some studies have found that sheep living at high altitudes experience long-term low-pressure hypoxia and reduced OS status, causing a high expression of VEGF in ovarian tissue. Furthermore, the use of antioxidant vitamins can reduce OS levels, but this effect is limited [35]. The MAPK cascade is one of the most important pathways in eukaryotic signal transduction networks and plays a key role in the regulation of gene expression and cytoplasmic function [36]. The impact of environmental pollutants such as industrial waste, has always been a concern on ovarian function. Bisphenol (BHPF), a major chemical component in many industrial products, has been found to be able to increase OS levels in mouse oocytes and reduce p-MAPK protein level, which in turn disrupts meiotic spindle assembly, inhibits oocyte maturation and ultimately affects ovarian function [37]. Therefore, we have speculated that EC may be an effective treatment for POI by regulating the expression of AKT1 to improve OS status.

**Figure 9:**
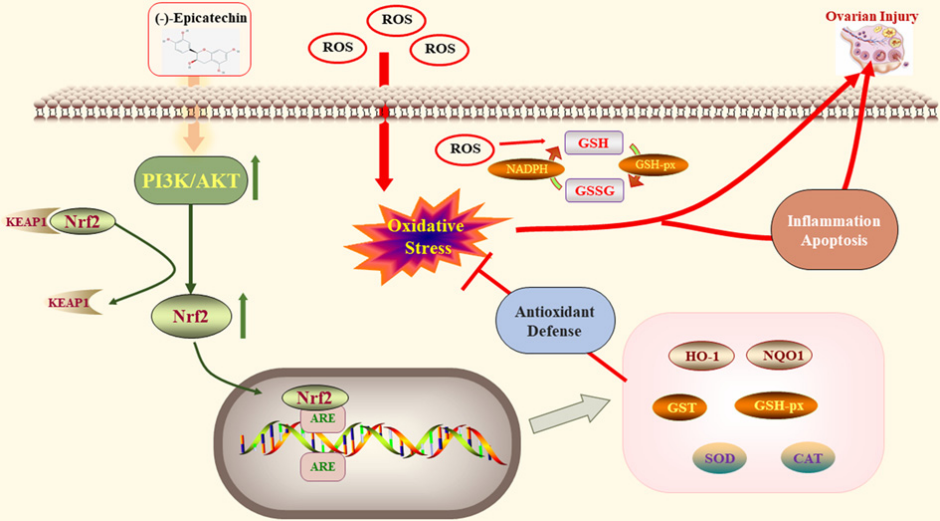
Demonstration of the internal antioxidant mechanism of EC in treatment of POI

Phosphatidylinositol 3-kinase (PI3K), accompanied by its vital downstream modulator AKT, is responsible for the formation of PI3K/AKT signaling pathway [38]. This pathway is a key regulator of many cellular processes and biological activities including cell proliferation, survival, growth, motility, cytoskeletal restructuring and metabolism, exerting these effects through multiple downstream targets [39,40]. Increasingly, studies have found that PI3K/AKT is the key regulatory pathway involved in ovarian function and it can regulate the resting state of primordial follicles, as well as their activation and survival. It can also induce proliferation and differentiation in granulosa cells, as well as meiosis and maturation of oocytes [41,42]. It is one of the main non-gonadotropin associated signaling pathways involved in insulin regulation, which can maintain the normal reproductive life time of the ovary [43]. Nrf2 is a leucine zipper transcription factor and one of the most important molecules downstream of AKT. As an important component of the antioxidant defense mechanism, Nrf2 plays a crucial role as a core sensor of stress, subsequent to cellular oxidative damage [44]. Under normoxia, Nrf2 is inactive as it is present in combination with Keap1. However, after cellular exposure to OS, this complex becomes dissociated and Nrf2 becomes translocated to the nucleus, where it combines with genomic antioxidant response elements (AREs) to promote the expression of a series of downstream antioxidant enzymes and detoxification factors, such as HO-1, NQO1 and SOD [45]. The activation of the PI3K/AKT pathway can promote the dissociation of Keap1–Nrf2 and the translocation of Nrf2 to the nucleus [46]. However, *in vitro* experiments have also found that EC can directly activate Nrf2 to exert its antioxidant effects [47]. Therefore, this study used ovarian granulosa cells to confirm the antioxidant effect of EC on treating POI through the PI3K/AKT/Nrf2 signaling pathway. Granulosa cells surround oocytes and play a key role in the regulation of the follicular fluid microenvironment, follicular growth and atresia [48]. H_2_O_2_, a kind of potent oxidant, is the most classic and most widely employed reagent in the establishment of various types of OS cell models, such as KGN cells and bovine granulosa cells [49,50]. Our results suggested that the mRNA levels and protein expression of PI3K, AKT and Nrf2 in the H_2_O_2_ treated group were significantly reduced. However, after the intervention of EC, there was a significant recovery and this effect appeared to be dose dependent. Downstream factors regulated by Nrf2 include stress and antioxidant genes and genes related to enzymes involved in cellular detoxification, including HO-1 and NQO1. In the present study, the mRNA and protein expression levels of HO-1 and NQO1 were significantly decreased in the model group, indicating that the Nrf2-ARE transduction pathway may be activated in granulosa cells to prevent ovarian OS damage. Furthermore, EC can up-regulate the mRNA and protein expression levels of HO-1 and NQO1 in a dose-dependent manner, suggesting that EC could exert a protective effect on granulosa cells through activation of the PI3K/AKT/Nrf2 pathway. NADPH is an important reducing coenzyme and works as an important hydrogen donor in cells by maintaining the reduced state of GSH and removing excessive oxidation products in cells [51]. In this study, we found that NADPH, GSH and GSSG were all detectable, and EC in medium-dose demonstrated its optimal antioxidant effects [52]. However, it was unclear why no tendency was seen to maintain a dose-dependent trend with the high-dose group. So we speculate that this may be related to the limited number of enzymes associated with the oxidative system in our cells and further investigations are needed. eNOS is an endothelial isoform of NO synthase, and its decoupling is an important mechanism leading to an increasing ROS levels [53]. In this study, protein levels of eNOS were found to be significantly increased in the H_2_O_2_ group, but this was attenuated by EC. SOD is an important component of the antioxidant enzyme cascade in biological systems and its detection in the present study confirmed a role for EC as an antioxidant. Therefore, we can conclude that EC has the ability to reduce the OS status of granulosa cells through the regulation of the PI3K/AKT/Nrf2 signaling pathway and thereby alleviate POI. This molecular pathway is depicted in Figure 9.

Our study had one main limitation that we only used *in vitro* experiments to verify the therapeutic effect of EC in POI instead of a combinatorial approach using *in vivo* experiments also. Therefore, in our follow-up experimental studies, we will look at the efficacy of EC in the treatment of POI by using both *in vivo* and *in vitro* models.

## Conclusion

The incidence of POI continues to increase significantly worldwide, and the OS status in ovaries appears to be an important pathological factor. EC, as a type of polyphenol with strong antioxidative effects, has been elucidated its therapeutic effects in other diseases gradually. In the present study, we employed a combination of network pharmacology and *in vitro* assays to explore the cellular mechanisms of EC against POI. A total of 70 potential targets for EC were obtained, of which, AKT1, VEGFA, CASP3 and IL6 represented important candidate targets. Our KEGG results showed that the common targets were significantly enriched in the PI3K/AKT, TNF and MAPK signaling pathways. Furthermore, critical cellular experiments provided evidence for a role for EC in an H_2_O_2_-mediated OS model in ovarian granulosa cells by activation of the PI3K/AKT/Nrf2 signaling pathway. In summary, EC has the ability to down-regulate elevated OS level through the PI3K/AKT/Nrf2 signaling pathway and represents a potential novel treatment for POI.

## Data Availability

The datasets used and/or analyzed during the current study are available from the corresponding author on reasonable request.

## Abbreviations

AKT: protein kinase B
BATMAN-TCM: Bioinformatics Analysis Tool for Molecular Mechanism of Traditional Chinese Medicine
BP: biological process
CCK-8: cell counting kit 8
DAVID: Database for Annotation, Visualization and Integrated Discovery
EC: (-)-Epicatechin
FBS: fetal bovine serum
GO: Gene Ontology
GSH: reduced glutathione
GSSG: oxidized glutathione
HO-1: heme oxygenase 1
KEGG: Kyoto Encyclopedia of Genes and Genomes
MF: molecular function
NADPH: nicotinamide adenine dinucleotide phosphate
NQO1: NADH quinone dehydrogenase 1
Nrf2: nuclear factor erythroid 2-related factor 2
OMIM: Online Mendelian Inheritance in Man
OS: oxidative stress
PI3K: phosphatidylinositol 3-kinase
POI: premature ovarian insufficiency
PPI: protein–protein interaction
SOD: superoxide dismutase
TCMSP: Traditional Chinese Medicine System Pharmacology Database
